# Catalase Mediates the Inhibitory Actions of PPARδ against Angiotensin II-Triggered Hypertrophy in H9c2 Cardiomyocytes

**DOI:** 10.3390/antiox10081223

**Published:** 2021-07-29

**Authors:** Jung Seok Hwang, Jinwoo Hur, Won Jin Lee, Jun Pil Won, Hyuk Gyoon Lee, Dae-Seog Lim, Eunsu Kim, Han Geuk Seo

**Affiliations:** 1 College of Sang-Huh Life Sciences, Konkuk University, 120 Neungdong-ro, Gwangjin-gu, Seoul 05029, Korea; mathking83@hanmail.net (J.S.H.); wlsdn91@konkuk.ac.kr (J.H.); Windfall@konkuk.ac.kr (W.J.L.); wjp0505@konkuk.ac.kr (J.P.W.); krci-12@daum.net (H.G.L.); np-gennao@hanmail.net (E.K.); 2 Department of Biotechnology, CHA University, 355 Pangyo-ro, Bundang-gu, Seongnam 13488, Korea; dslim@cha.ac.kr

**Keywords:** angiotensin II, catalase, hypertrophy, peroxisome proliferator-activated receptor δ, reactive oxygen species

## Abstract

Hypertrophy of myocytes has been implicated in cardiac dysfunctions affecting wall stress and patterns of gene expression. However, molecular targets potentially preventing cardiac hypertrophy have not been fully elucidated. In the present study, we demonstrate that upregulation of catalase by peroxisome proliferator-activated receptor δ (PPARδ) is involved in the anti-hypertrophic activity of PPARδ in angiotensin II (Ang II)-treated H9c2 cardiomyocytes. Activation of PPARδ by a specific ligand GW501516 significantly inhibited Ang II-induced hypertrophy and the generation of reactive oxygen species (ROS) in H9c2 cardiomyocytes. These effects of GW501516 were almost completely abolished in cells stably expressing small hairpin (sh)RNA targeting PPARδ, indicating that PPARδ mediates these effects. Significant concentration and time-dependent increases in catalase at both mRNA and protein levels were observed in GW501516-treated H9c2 cardiomyocytes. In addition, GW501516-activated PPARδ significantly enhanced catalase promoter activity and protein expression, even in the presence of Ang II. GW501516-activated PPARδ also inhibited the expression of atrial natriuretic peptide (ANP) and B-type natriuretic peptide (BNP), which are both marker proteins for hypertrophy. The effects of GW501516 on the expression of ANP and BNP were reversed by 3-amino-1,2,4-triazole (3-AT), a catalase inhibitor. Inhibition or downregulation of catalase by 3-AT or small interfering (si)RNA, respectively, abrogated the effects of PPARδ on Ang II-induced hypertrophy and ROS generation, indicating that these effects of PPARδ are mediated through catalase induction. Furthermore, GW501516-activated PPARδ exerted catalase-dependent inhibitory effects on Ang II-induced hypertrophy by blocking p38 mitogen-activated protein kinase. Taken together, these results indicate that the anti-hypertrophic activity of PPARδ may be achieved, at least in part, by sequestering ROS through fine-tuning the expression of catalase in cardiomyocytes.

## 1. Introduction

An increase in the size of cardiomyocytes characterizes cardiac hypertrophy, which is attributed to adaptive responses that compensate for increased ventricular wall stress resulting from hemodynamic overload [1]. Diverse stimuli, including mechanical stress and neurohumoral factors such as angiotensin II (Ang II), tumor necrosis factor-α, phenylephrine, and endothelin-1, are associated with the progression of cardiac hypertrophy [1,2]. These factors also trigger the production of cardiac reactive oxygen species (ROS), which are implicated as signaling molecules in the development of cardiac hypertrophy [3]. ROS can directly or indirectly activate downstream signaling cascades, especially mitogen-activated protein kinases (MAPKs), including p38, extracellular signal-regulated kinase (ERK), and Jun N-terminal kinase (JNK), which may eventually effect the onset of cardiac hypertrophy [4]. Many studies indicate that blocking cardiac ROS production can have a direct effect on arterial blood pressure and myocardial cells, including cardiomyocyte growth and hypertrophy [5,6]. Although the pathophysiology of hypertrophy is complex and multifactorial, and involves several cellular and molecular systems, insights into the molecular background of cardiac hypertrophy are essential to protect the myocardium against pathological remodeling. Therefore, blocking the excess production of ROS may be a strategy for preventing the progression of cardiac hypertrophy and slowing down the ultimate onset of heart failure.

Peroxisome proliferator-activated receptor (PPAR) δ is a ligand-activated nuclear receptor that elicits diverse biological activities by regulating the expression of its target genes [7]. Recent studies showed that activation of PPARδ by a specific ligand exerts protective effects through anti-inflammatory or anti-senescence mechanisms in the vasculature [8,9]. This nuclear receptor also exerts atheroprotective effects in an Ang II-accelerated animal model of atherosclerosis [8], and ligand-activated PPARδ also modulates oxidative stress in cardiovascular cells by regulating the expression of target genes such as manganese superoxide dismutase, glutathione peroxidase, thioredoxin, heme oxygenase-1, and thrombospondin-1 [10,11]. Furthermore, we previously demonstrated that ligand-activated PPARδ counteracts ROS production in vascular cells to inhibit cellular senescence induced by Ang II [9]. It has also been demonstrated that PPARδ activation inhibits cardiac hypertrophy induced by Ang II and hyperglycemia through suppression of the intracellular Ca^2+^ signaling pathway and free radical production in cultured H9c2 cardiomyocytes [12,13]. Based on its ability to protect the cardiovasculature [8,9,10,11,12], investigating the potential mechanisms of PPARδ in the development of cardiac hypertrophy could prove fruitful from a therapeutic perspective.

Emerging evidence indicates that excessive accumulation of ROS caused by upregulation of ROS-producing enzymes or downregulation of antioxidant enzymes under myocardial stress can exacerbate overall oxidative stress in myocardium under pathological conditions [14]. Catalase is a ubiquitous antioxidant enzyme that specifically converts hydrogen peroxide into water and oxygen molecules, and thereby reduces the cellular accumulation of ROS [15]. Our previous study demonstrated that activation of PPARδ by the specific ligand GW501516 reduces oxidative stress in vascular cells by reducing ROS accumulation through regulating the expression of target genes [10,11,16,17]. However, it remains unclear whether PPARδ can prevent cardiac hypertrophy simply by reducing ROS accumulation. Thus, the present work aimed to examine the effect of PPARδ activation on catalase expression to determine whether there is a mechanistic link between vasoactive peptide Ang II-associated cardiac hypertrophy and PPARδ-mediated upregulation of catalase. Our results show that activation of PPARδ by the specific ligand GW501516 upregulates the expression of catalase in H9c2 cardiomyocytes. Furthermore, GW501516-activated PPARδ reduced ROS accumulation, thereby relieving Ang II-induced hypertrophy of myocardial cells. Although PPARδ elicited beneficial effects in opposition to oxidative stress-induced vascular damage by inhibiting ROS generation in diverse cell lineages [9,10,11,12,13,14,17], little is known about actual effector molecules involved in this mechanism. We identified catalase as an effector molecule in the PPARδ-mediated reduction of ROS accumulation, thereby preventing cardiac hypertrophy induced by Ang II.

## 2. Materials and Methods

### 2.1. Material

Polyclonal rabbit anti-β-actin antibody, 3-(4,5-dimethylthiazol-2-yl)-2,5-diphenyltetrazolium bromide (MTT), and 3-amino-1,2,4-triazole (3-AT) were provided by Sigma-Aldrich Co. (St. Louis, MO, USA). Ang II, SB203580, SP600125, PD98059, and 2′,7′-dichlorofluorescein diacetate (H2DCF-DA) were provided by Calbiochem (La Jolla, CA, USA). Polyclonal rabbit anti-catalase antibody and GW501516 were provided by GeneTex (Irvine, CA, USA) and Enzo Life Sciences (Farmingdale, NY, USA), respectively. Monoclonal mouse antibodies specific for PPARδ and α-actinin conjugated with Alexa Fluor 488 were from Santa Cruz Biotechnology (Santa Cruz, CA, USA). Rabbit polyclonal antibodies specific for p38, extracellular signal-regulated kinase (ERK), c-Jun N-terminal kinase (JNK), phospho-p38, phospho-ERK, and phospho-JNK were obtained from Cell Signaling (Beverly, MA, USA).

### 2.2. Cell Culture

H9c2 cells (rat cardiomyocytes) were obtained from Korean Cell Line Bank (KCLB number 21446; Seoul, Korea) and maintained in Dulbecco’s modified eagle’s medium (DMEM) supplemented with 10% fetal bovine serum (FBS) in the presence of 1% antibiotics (100 U/mL penicillin and 100 μg/mL streptomycin) at 37 °C with 5% CO_2_.

### 2.3. MTT Assay

To determine the toxicity of GW501516 on cell viability, 2 × 10^4^ H9c2 cells were plated in 24-well plates and stimulated with various concentrations of GW501516 for 24 h. After treatment with MTT solution (final 0.1 mg/mL), cells were further incubated for 2 h. Following removal of medium, formazan crystals that were formed by mitochondrial dehydrogenase-mediated reduction of MTT in living cells were dissolved in acidified isopropanol and the absorbance was spectrophotometrically measured at 570 nm.

### 2.4. Immunofluorescence Staining

H9c2 cells plated at a density of 1 × 10^5^ cells in a 35 mm culture dish were stimulated with the indicated reagents. Following brief washing with chilled phosphate-buffered saline (PBS), cells were fixed with 4% paraformaldehyde solution for 10 min at 4 °C. After washing twice with PBS, cells were incubated in PBS containing 0.3% Triton X-100 and 3% bovine serum albumin (BSA) for 60 min at room temperature, and then reacted with α-actinin antibody conjugated with Alexa Fluor 488 (1:200) at 4 °C overnight. To visualize nuclei, cells were stained with propidium iodide for 5 min, and immunofluorescence images were captured on a Nikon Eclipse Ti2 inverted fluorescent microscope (Nikon, Tokyo, Japan). ImageJ software was used to evaluate the cell surface area based on α-actinin-positive staining.

### 2.5. Measurement of Intracellular Reactive Oxygen Species (ROS)

H2DCF-DA, a fluorescent probe, was used to assess levels of intracellular ROS. Briefly, H9c2 cells plated at a density of 1 × 10^5^ cells on a 35 mm glass-bottomed dish (SPL Life Sciences, Seoul, Korea) were stimulated with reagents for the indicated times and then treated with 10 µM H2DCF-DA for 30 min. After brief washing with PBS, levels of intracellular ROS corresponding to green fluorescence were detected at 520 nm using a Nikon Eclipse Ti2 inverted fluorescence microscope.

### 2.6. Gene Silencing with Small Interfering RNA (siRNA)

H9c2 cells at a density of 1 × 10^5^ cells were transfected with siRNA recognizing scrambled nonspecific sequences (Ambion, Austin, TX, USA) or rat catalase siRNA recognizing the sequences of catalase mRNA designed (Table S1) using SuperFect (Qiagen, Valencia, CA, USA) in serum-containing medium, as described previously [18]. The siRNA recognizing catalase was synthesized by Bioneer (Daejeon, Korea). Following transfection for 6 h, cells were treated with complete fresh medium and grown for an additional 18 h, and then treated with reagents for the indicated time periods. The efficiency of gene silencing was verified by Western blotting.

### 2.7. Construction of H9c2 Cells Stably Expressing Short Hairpin (sh)PPARδ

PPARδ-silenced H9c2 cells were constructed by transduction of lentiviral pLKO.1-puro PPARδ-target shRNA particles or a pLKO.1-puro non-Target shRNA Particles (Sigma-Aldrich, St. Louis, USA). Following incubation for 24 h, transduced cells were incubated with 2 μg/mL puromycin for selection, and PPARδ silencing was verified by Western blotting.

### 2.8. Western Blot Analysis

H9c2 cells at a density of 2.5 × 10^5^ cells exposed to the indicated reagents were lysed in PRO-PREP Protein Extraction Solution (iNtRON Biotechnology, Seoul, Korea), and an aliquot of the lysate was resolved by sodium dodecyl sulfate polyacrylamide gel electrophoresis (SDS-PAGE) and then transferred onto a Hybond-P+ polyvinylidene difluoride (PVDF) membrane (Amersham Biosciences, Buckinghamshire, UK). Following blocking with 5% nonfat milk at 4 °C overnight, membranes were incubated with the indicated specific antibodies at 4 °C overnight. Membranes were then reacted with secondary antibody (1:5,000) at room temperature for 1 h. Following rinsing in TBS containing 0.1% Tween 20, the signals were detected using WesternBright ECL (Advansta Inc., Menlo Park, CA, USA).

### 2.9. Real-Time PCR

Total RNA isolated by TRIzol reagent (Invitrogen, Carlsbad, CA, USA) was synthesized into cDNA using a TOPscript RT DryMIX kit (Enzynomics, Seoul, Korea) to assess mRNA levels, as described previously [19]. Real-time PCR was conducted using equal amounts of cDNA in a 10 μL reaction containing primers and 1× Real-Time PCR mix (Solgent, Daejeon, Korea), followed by 50 cycles of PCR amplification (10 s at 95 °C, 10 s at 58.3 °C, and 10 s at 72 °C). The primer sequences used are presented in Table S1. The expression of the target gene was determined as the fold change relative to GAPDH using the ΔΔCT method [19].

### 2.10. Construction of the pGL3-Catalase Luciferase Reporter Plasmid and the Reporter Gene Assay

Genomic DNA isolated from human dermal fibroblasts was used as a template to amplify the catalase promoter region (−3029 to +10). Primers including KpnI and BglII restriction sites were designed based on the sequence of the 5′-untranslated region of catalase (GenBank accession number NC_000011). Sense and antisense oligonucleotides (Table S1) were employed, and the PCR product was digested with BglII and KpnI and ligated into the similarly digested pGL3-Basic luciferase reporter vector (Promega, Madison, WI, USA). For reporter gene assays, 2 × 10^4^ H9c2 cells were co-transfected with pGL3-catalase and pSV β-Gal (SV40 β-galactosidase expression vector, Promega) using SuperFect (Qiagen). After incubation for 24 h, cells were exposed to indicated reagents and lysed in luciferase reporter lysis buffer (Promega), and promoter activity was determined using lysates on a Microlumat Plus LB96V instrument (EG&G Berthold, Bad Wildbad, Germany), as described previously [20].

### 2.11. Statistical Analysis

Data are presented as mean ± standard error (SE). Statistical significance between groups was assessed using one-way analysis of variance (ANOVA) followed by Tukey–Kramer tests. Single comparisons with control samples were performed using two-tailed Student’s paired *t*-tests. All results were tested by Kolmogorov–Smirnov test for normality. A Kruskal–Wallis H test or Mann–Whitney U test was used to analyze data that were not normally distributed. Statistical analysis was performed using Statistical Package for Social Sciences (SPSS, Version 18; IBM Corp., New York, NY, USA).

## 3. Results

### 3.1. GW501516-Activated PPARδ Inhibits Ang II-Triggered Hypertrophy and ROS Generation in H9c2 Cells

To select the optimal dose range of GW501516 for H9c2 cells, we determined cell viability using MTT assays. When cells were exposed to various concentrations of GW510156 (0, 0.5, 1, 10, 50, and 100 nM) for 24 h, cytotoxicity was not observed at any of the dosages (Figure S1). Thus, we chose 100 nM GW501516 as an optimal dosage for subsequent studies on H9c2 cells.

**Figure 1:**
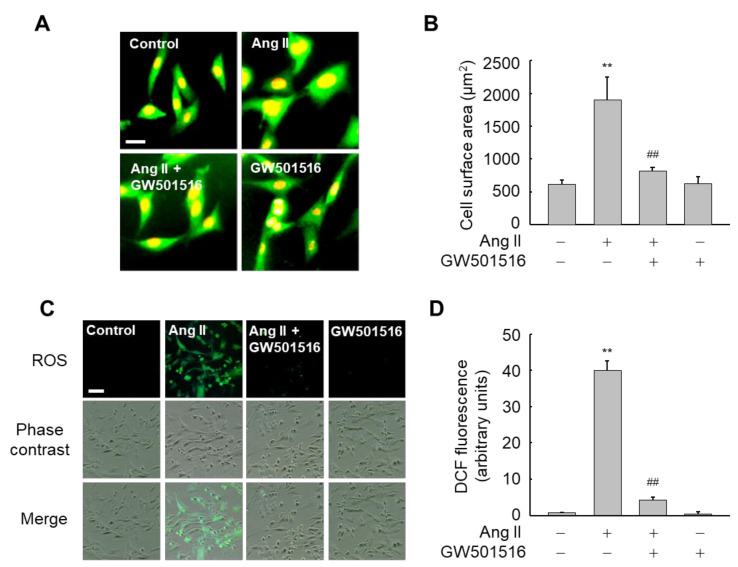
GW501516-activated PPARδ attenuates Ang II-induced cellular hypertrophy and ROS production in H9c2 cardiomyocytes. (**A**,**B**) Cells pretreated with 100 nM GW501516 for 12 h were incubated in the presence or absence of 100 nM Ang II for 24 h. Immunofluorescence assays were then performed using α-actinin antibody (**A**), and cell surface area was quantified using an image analyzer and then plotted (**B**). Nuclei were stained with propidium iodide. (**C**,**D**) Cells pretreated with 100 nM GW501516 for 12 h were incubated with 100 nM Ang II for 2 h. Intracellular ROS accumulation was then analyzed by fluorescence microscopy using CM-H2DCF-DA (10 μM), a peroxide-sensitive dye (**C**), and the fluorescence intensity was quantified (**D**). Bars = 100 μm. Representative images from four independent experiments are shown. Results are expressed as mean ± standard error (SE) (*n* = 4; ** *p* < 0.01 compared with the untreated group; ^##^ *p* < 0.01 compared with the Ang II-treated group).

Since Ang II has been implicated in cardiac hypertrophy [21], we assessed whether activation of PPARδ might affect cell surface area (CSA) in H9c2 cells exposed to Ang II. Cells treated with Ang II showed a significant increase in CSA, an indicator for cellular hypertrophy, relative to untreated control cells. By contrast, the increase in CSA was significantly reduced in the presence of GW501516, a PPARδ-specific ligand, suggesting that PPARδ is involved in the inhibition of Ang II-induced hypertrophy (Figure 1A,B). In addition, since Ang II is also known to produce ROS in cardiomyocytes [21], we also investigated the effects of GW501516 on production of ROS in H9c2 cells exposed to Ang II. Whereas Ang II dramatically induced ROS levels, pretreatment with GW501516 significantly inhibited ROS levels induced by Ang II (Figure 1C,D).

**Figure 2:**
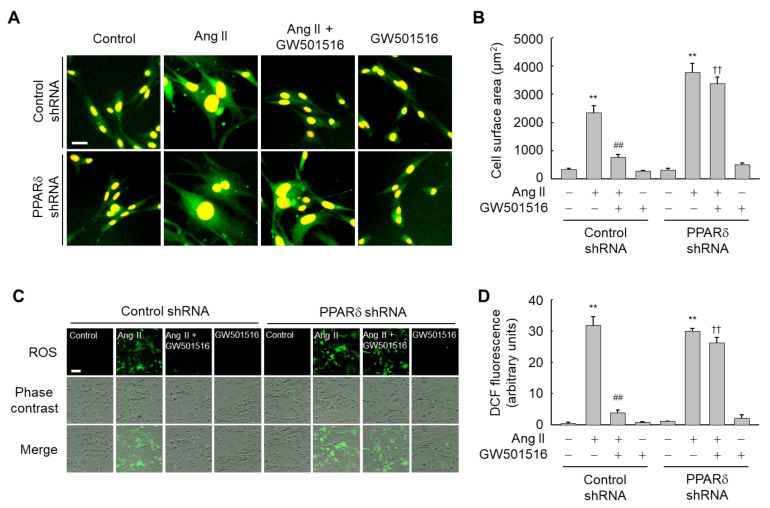
RNAi-mediated knockdown of PPARδ abolishes the effects of GW501516 on Ang II-induced hypertrophy and ROS production in H9c2 cardiomyocytes. Cells stably expressing PPARδ shRNA or scrambled control shRNA were pretreated with 100 nM GW501516 or vehicle (DMSO) for 12 h, and then exposed to 100 nM Ang II. Following incubation for 24 h (for hypertrophy detection) or 2 h (for ROS detection), immunofluorescence assays were performed using α-actinin antibody (**A**), and cell surface area was quantified using an image analyzer and then plotted (**B**). Intracellular ROS accumulation was analyzed by fluorescence microscopy using 10 μM CM-H2DCF-DA (**C**), and the fluorescence intensity was quantified (**D**). Representative images from four independent experiments are shown. Bars = 100 μm. Results are expressed as mean ± standard error (SE) (*n* = 4; ** *p* < 0.01 compared with the untreated group; ^##^ *p* < 0.01 compared with the Ang II-treated group; ^††^ *p* < 0.01 compared with the Ang II plus GW501516-treated control shRNA group).

To clarify the roles of PPARδ in the blockage of Ang II-induced hypertrophy and ROS generation, the effect of GW501516 was determined in H9c2 cells stably expressing shRNA against PPARδ or a nonspecific control shRNA. Levels of PPARδ in H9c2 cells were markedly reduced upon transfection with PPARδ-targeting shRNA, whereas the control shRNA had no effect on PPARδ levels (Figure S2). As expected, the GW501516-mediated reductions in hypertrophy and ROS generation were reversed in cells transfected with PPARδ-targeting shRNA, indicating that the effects of GW501516 on ROS generation and cellular hypertrophy are dependent on PPARδ (Figure 2A–D).

### 3.2. GW501516-Activated PPARδ Induces the Expression of Catalase in H9c2 Cells

**Figure 3:**
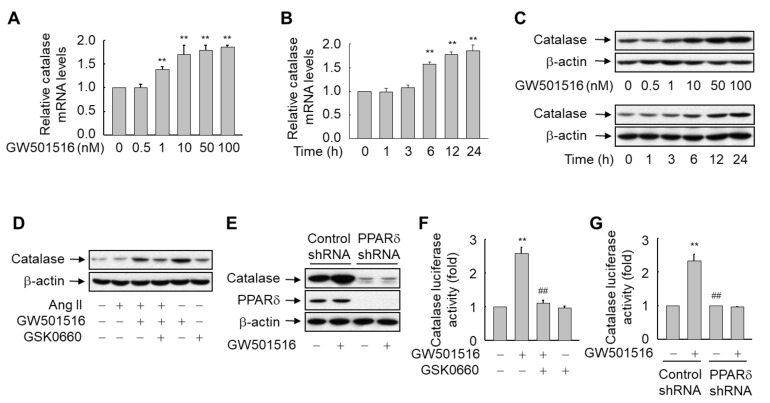
A PPARδ ligand GW501516 upregulates the expression of catalase at both mRNA and protein levels in H9c2 cardiomyocytes. (**A**–**C**) Cells were incubated for 24 h with various concentrations of GW501516 (**A** and upper panels of **C**) or exposed to 100 nM GW501516 for the times indicated (**B** and lower panels of **C**). (**D**) Cells were pretreated with 100 nM GW501516 and/or 1 μM GSK0660 for 12 h, and then exposed to 100 nM Ang II for 24 h. (**E**) Cells stably expressing PPARδ shRNA or scrambled control shRNA were treated with or without 100 nM GW501516 for 24 h. Expression levels of catalase mRNA and protein were analyzed by real-time PCR (**A**,**B**) and Western blotting (**C**–**E**). (**F**,**G**) Cells pretreated with 1 μM GSK0660 for 1 h (**F**) or stably expressing shRNA against PPARδ or scrambled control sequences (**G**) were transfected with catalase luciferase reporter plasmid (1.5 µg) and pSV-β-gal (0.5 µg), and then exposed to 100 nM Ang II. After incubation for 24 h, cells were harvested, and cell lysates were subjected to reporter gene assays. Results are expressed as mean ± standard error (SE) of 3 to 4 independent experiments (** *p* < 0.01 compared with the untreated group; ^##^ *p* < 0.01 compared with the Ang II-treated group).

Exposure of H9c2 cells to GW501516 induced the expression of catalase in a dose and time-dependent manner. Maximum levels of catalase mRNA were obtained after 24 h of exposure to 1–100 nM GW501516 (Figure 3A). When cells were treated with 100 nM GW501516, the increase in catalase mRNA was significant at 6 h and continued for up to 24 h (Figure 3B). Catalase protein levels were also elevated at 6 h after incubation with 100 nM GW501516, and this continued for up to 24 h. Maximum protein abundance was reached after a 24 h incubation with 10–100 nM GW501516 (Figure 3C).

To further define the role of PPARδ in the upregulation of catalase, we examined the effect of GW501516 in H9c2 cells stably expressing shRNA targeting PPARδ or pretreated with GSK0660, a specific inhibitor of PPARδ [22]. GSK0660-mediated inhibition or shRNA-mediated knockdown of PPARδ reversed the expression pattern of catalase induced by the PPARδ agonist, even in the presence of Ang II, which had no effect on the expression of catalase (Figure 3D,E and Figure S3). These results clearly indicate that PPARδ is involved in the regulation of catalase expression in H9c2 cells.

To determine whether the PPARδ-mediated expression of catalase occurs at the level of transcription, a reporter gene assay was carried out using a luciferase reporter construct driven by the murine catalase promoter. GW501516-activated PPARδ significantly enhanced the promoter activity of catalase, which is consistent with the increase in catalase at both transcript and protein levels. By contrast, this effect was completely abolished in the presence of PPARδ shRNA or GSK0660, suggesting that PPARδ induces the expression of catalase at the transcription level (Figure 3F,G).

### 3.3. GW501516-Activated PPARδ Regulates the Expression of Marker Proteins Linked to Ang II-Induced Hypertrophy of H9c2 Cardiomyocytes

**Figure 4:**
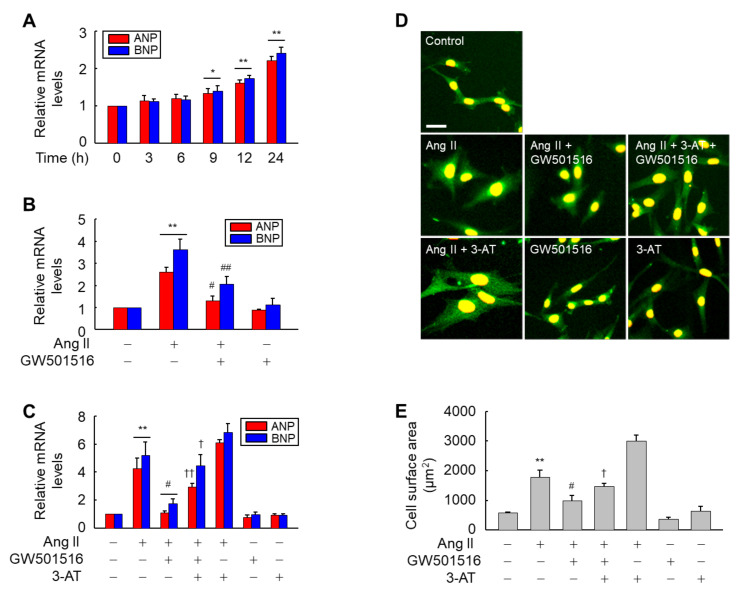
GW501516-activated PPARδ attenuates the Ang II-induced expression of marker proteins and hypertrophy through catalase in H9c2 cardiomyocytes. (**A**) Cells were incubated with 100 nM GW501516 for the times indicated. (**B**) Cells were pretreated with 100 nM GW501516 for 12 h, and then exposed to 100 nM Ang II for 24 h. (**C**–**E**) Cells pretreated with 100 nM GW501516 and/or 30 mM 3-amino-1,2,4-triazole (3-AT) for 12 h were treated with 100 nM Ang II for 24 h. The mRNA levels of ANP and BNP were analyzed by real-time PCR (**A**–**C**). Immunofluorescence assays were performed using α-actinin antibody (**D**) and cell surface area was quantified using an image analyzer and plotted (**E**). Nuclei stained with propidium iodide. Representative images from four independent experiments are shown. Bars = 100 µm. Results are expressed as mean ± standard error (SE) of 3 to 4 independent experiments (** *p* < 0.01, * *p* < 0.05 compared with the untreated group; ^##^ *p* < 0.01, ^#^ *p* < 0.05 compared with the Ang II-treated group; ^††^ *p* < 0.01, ^†^ *p* < 0.05 compared with the Ang II plus GW501516-treated group).

ANP and BNP are considered marker proteins in hypertrophy induced by Ang II [23]. Therefore, we examined whether these proteins are associated with PPARδ-mediated regulation of hypertrophy of H9c2 cardiomyocytes induced by Ang II. The expression of both ANP and BNP was increased upon exposure to Ang II, evident at 9 h, and elevation continued for up to 24 h in a time-dependent manner (Figure 4A). This Ang II-induced increase in the mRNA levels of both ANP and BNP was significantly reduced by treatment with GW501516 (Figure 4B). On the other hand, GW501516-mediated suppression of both transcripts was significantly reversed by addition of 3-AT, a specific inhibitor of catalase [24], suggesting that catalase mediates the anti-hypertrophic effects of PPARδ in H9c2 cardiomyocytes treated with Ang II (Figure 4C).

**Figure 5:**
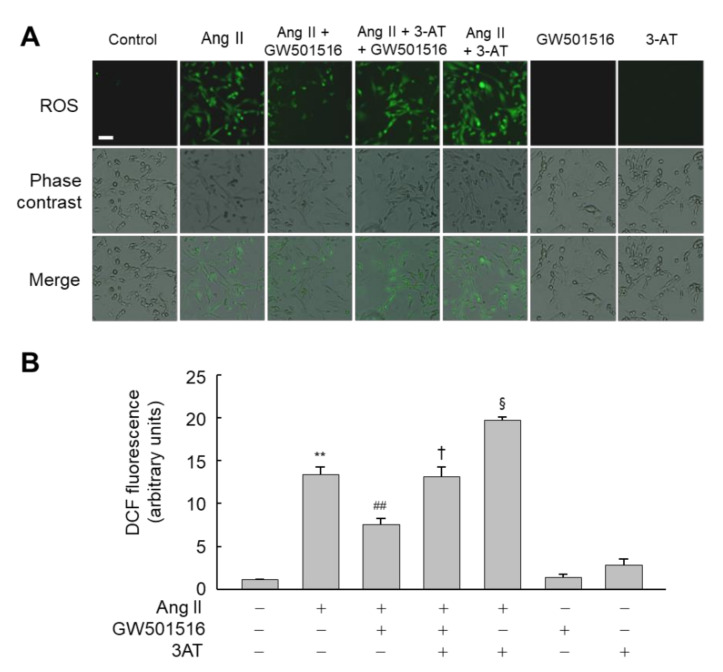
GW501516-activated PPARδ suppresses Ang II-induced ROS accumulation through catalase in H9c2 cardiomyocytes. Cells were pretreated with 100 nM GW501516 and/or 30 mM 3-AT for 12 h, and then exposed to 100 nM Ang II for 2 h. Intracellular ROS accumulation was analyzed by fluorescence microscopy using 10 μM CM-H2DCF-DA (**A**), and the fluorescence intensity was quantified (**B**). Representative images from four independent experiments are shown. Bars = 100 μm. Results are expressed as mean ± standard error (SE) (*n* = 4; ** *p* < 0.01 compared with the untreated group; ^##^ *p* < 0.01 compared with the Ang II-treated group; ^†^ *p* < 0.05 compared with the Ang II and GW501516-treated group; ^§^ *p* < 0.05 compared with the Ang II, GW501516, and 3-AT-treated group).

To further clarify the functional significance of catalase induction by PPARδ, we assessed the impact of 3-AT in the PPARδ-mediated suppression of hypertrophy induced by Ang II. In line with the expressional regulation of marker proteins for hypertrophy, 3-AT-mediated inhibition of catalase activity significantly counteracted the GW501516-mediated inhibitory action of PPARδ in the CSA of H9c2 cardiomyocytes treated with Ang II (Figure 4D,E). These results suggest that PPARδ regulates Ang II-induced hypertrophy by modulating the expression of catalase. Next, we evaluated the effect of catalase inhibition by 3-AT on ROS accumulation in H9c2 cardiomyocytes, because ROS is implicated in Ang II-induced hypertrophy [25]. When cells were pretreated with 3-AT in the presence of Ang II and GW501516, 3-AT-mediated inhibition of catalase activity reversed the effect of GW501516 to repress ROS generation, suggesting that PPARδ-mediated suppression of ROS production is dependent on catalase activity (Figure 5A,B).

### 3.4. Downregulation of Catalase Abrogates the Effects of PPARδ on Hypertrophy and ROS Production

**Figure 6:**
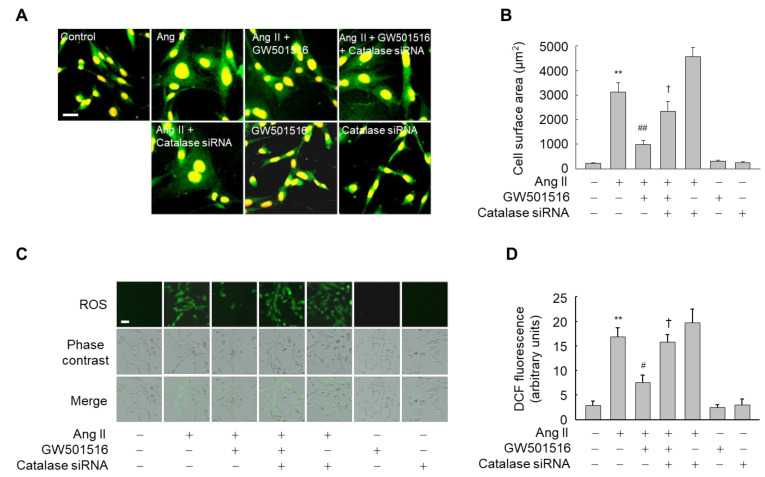
Knockdown of catalase by siRNA abrogates the effects of GW501516 on hypertrophy and ROS production triggered by Ang II in H9c2 cardiomyocytes. Cells transfected with catalase siRNA (200 nM) or control siRNA (200 nM) for 24 h were pretreated with 100 nM GW501516 or vehicle (DMSO) for 12 h, and then stimulated with 100 nM Ang II. Following incubation for 24 h (hypertrophy detection) or 2 h (ROS detection), immunofluorescence assays were performed using α-actinin antibody (**A**), and cell surface area was quantified using an image analyzer and then plotted (**B**). Intracellular ROS accumulation was analyzed by fluorescence microscopy using 10 μM CM-H2DCF-DA (**C**), and the fluorescence intensity was quantified (**D**). Representative images from four independent experiments are shown. Bars indicate 100 µm. Results are expressed as mean ± standard error (SE) (*n* = 4; ** *p* < 0.01 compared with the untreated group; ^##^ *p* < 0.01, ^#^ *p* < 0.05 compared with the Ang II-treated group; ^†^ *p* < 0.05 compared with the Ang II plus GW501516-treated group).

To further confirm the role of catalase in PPARδ-mediated suppression of hypertrophy and ROS generation stimulated by Ang II, the expression of catalase was knocked down by siRNA in H9c2 cardiomyocytes (Figure S4). GW501516 suppressed both Ang II-induced CSA and ROS generation in H9c2 cardiomyocytes, whereas siRNA-mediated knockdown of catalase significantly reversed the effect of GW501516 on both CSA and ROS accumulation in H9c2 cardiomyocytes exposed to Ang II (Figure 6). These findings indicate that PPARδ inhibits Ang II-induced hypertrophy and ROS generation through catalase.

### 3.5. GW501516-Activated PPARδ Attenuates Ang II-Induced Hypertrophy and ROS Generation by Inhibiting p38 MAPK Signaling in H9c2 Cardiomyocytes

**Figure 7:**
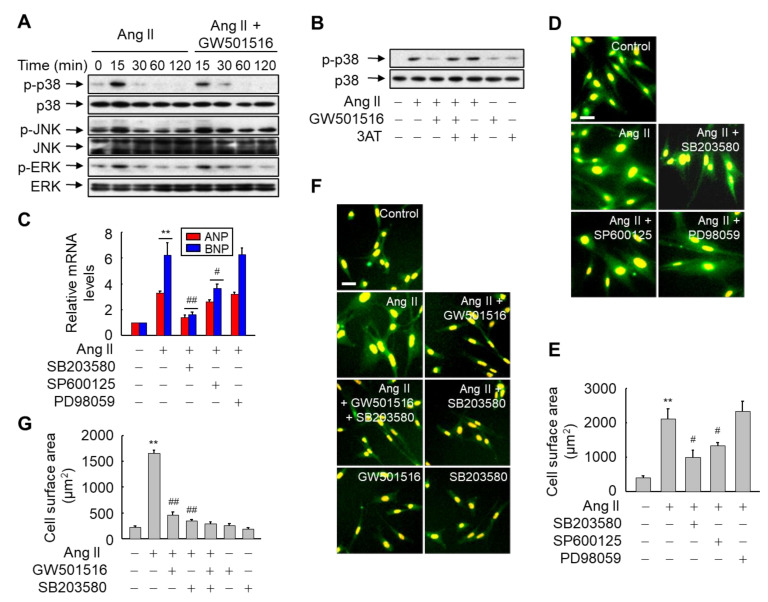
GW501516-activated PPARδ suppresses Ang II-triggered hypertrophy by inhibiting MAP kinase p38 in H9c2 cardiomyocytes. (**A**) Cells were exposed to 100 nM Ang II for the indicated times after pretreatment with 100 nM GW501516 or vehicle (DMSO) for 12 h. (**B**) Cells pretreated with 100 nM GW501516 and/or 30 mM 3-AT for 12 h were treated with 100 nM Ang II for 15 min. An aliquot of protein was immunoblotted using activation-specific antibodies, and parallel immunoblots were analyzed for total kinase levels. (**C**–**E**) Cells pretreated with 100 nM GW501516 and/or MAP kinase inhibitor 10 μM SB203580, 20 μM SP600125, or 10 μM PD98059 for 30 min were treated with 100 nM Ang II for 24 h. The expression levels of ANP and BNP, and hypertrophic changes, were analyzed via Western blotting (**C**) and immunofluorescence assays (**D**). Cell surface area was quantified using an image analyzer and plotted (**E**). (**F**,**G**) Cells pretreated with 100 nM GW501516 and/or 10 μM SB203580 for 12 h were exposed to 100 nM Ang II for 24 h. Immunofluorescence assays were performed (**F**), and cell surface area was quantified using an image analyzer and then plotted (**G**). Representative images from four independent experiments are shown. Bars = 100 μm. Results are expressed as mean ± standard error (SE) (*n* = 4; ** *p* < 0.01 compared with the untreated group; ^##^ *p* < 0.01, ^#^ *p* < 0.05 compared with the Ang II-treated group).

Since ROS act as second messengers that activate members of the MAPK family [26], we analyzed the involvement of MAPK pathways in Ang II-induced hypertrophy of H9c2 cardiomyocytes. In Ang II-stimulated cells, three MAPK cascades were immediately activated. Among these pathways, GW501516-activated PPARδ markedly inhibited Ang II-induced phosphorylation of p38 but not JNK or ERK pathways (Figure 7A). To verify the association of PPARδ-mediated catalase upregulation with the activation of p38 MAPK, cells were preincubated for 12 h with catalase inhibitor and/or GW501516, and then stimulated with Ang II for 15 min. GW501516 reduced Ang II-induced phosphorylation of p38, but the GW501516-mediated reduction was markedly reversed in the presence of 3-AT, indicating that PPARδ-mediated inhibition of p38 MAPK is involved in phenotypic changes caused by GW501516 in H9c2 cardiomyocytes (Figure 7B).

To further clarify the signaling pathways associated with Ang II-induced hypertrophy, we investigated the effects of the specific inhibitors of the three MAPK cascades in H9c2 cardiomyocytes exposed to Ang II. As shown in Figure 7C, the Ang II-induced increases in mRNA levels of ANP and BNP were significantly reduced in the presence of SB203580 (an inhibitor of the p38 pathway), and to a lesser extent in the presence of SP600125 (an inhibitor of the JNK pathway). PD98059 did not have such effects (an inhibitor of the ERK pathway). Similar effects of SB203580 and SP600125 were also observed for CSA in H9c2 cardiomyocytes treated with Ang II (Figure 7D,E). These results indicate that although both p38 and JNK signaling pathways can inhibit Ang II-mediated hypertrophy, only the p38-mediated signaling pathway is associated with PPARδ-mediated blocking of hypertrophy induced by Ang II. In addition, the p38 inhibitor, SB203580, reduced hypertrophy following Ang II stimulation to a similar extent to GW501516 (Figure 7F,G). However, treatment with GW501516 and SP600125 in combination did not yield results that differed from those of treatment with GW501516 alone, suggesting that the effects of PPARδ on the suppression of Ang II-induced hypertrophy are mediated via the p38 signaling pathway.

## 4. Discussion

Nuclear receptor PPARδ is a transcription factor known to modulate cellular functions by regulating the expression of diverse genes in the vasculature [7]. Although emerging evidence indicates that catalase is cardiovascular-protective, particularly against oxidative stress-associated cardiovascular disorders and Ang II-induced hypertrophy [27], molecular switches that modulate its expression or activity in cardiovascular cells are rarely reported. In the present study, we demonstrated that GW501516, a specific agonist of PPARδ, significantly inhibits Ang II-triggered hypertrophy and ROS production in H9c2 cardiomyocytes. Catalase, a known anti-hypertrophic factor [27], was significantly upregulated in H9c2 cardiomyocytes exposed to GW501516. Suppression of catalase expression or activity by siRNA or inhibitor 3-AT, respectively, antagonized the PPARδ-mediated inhibitory action against increased hypertrophic changes and ROS production in H9c2 cardiomyocytes.

The anti-hypertrophic activity of PPARδ is related to its ability to induce the activity of the intracellular antioxidant enzyme catalase in cardiomyocytes. The present findings are consistent with a previous report showing that ligand-activated PPARδ modulates the expression of antioxidant genes targeting ROS in vascular cells exposed to Ang II [9]. This antioxidant activity of PPARδ is directly linked to the inhibitory effect on hypertrophy of vascular smooth muscle cells stimulated by Ang II [28]. In fact, multiple studies have demonstrated that pharmacological activation of PPARδ by specific ligands can improve cardiac hypertrophy in cellular and animal models through mechanisms not yet fully elucidated [13,29,30,31]. By contrast, other reports have demonstrated that inducible conditional expression of PPARδ in cardiac endothelial cells leads to rapid cardiac angiogenesis and growth, including increased cardiomyocyte size [32]. However, most previous studies showed that PPARδ is cardioprotective, as demonstrated by in vivo and in vitro experiments showing reduced cardiomyocyte hypertrophy and myocardial injury due to ischemia/reperfusion [30,33]. Although the safety of the PPARδ activator GW501516 remains controversial because of its possible carcinogenic potential [34], the present results clearly demonstrate that PPARδ plays an important role in alleviating hypertrophy induced by Ang II in H9c2 cardiomyocytes. Accordingly, the present findings suggest a primary role for PPARδ as a new potential therapeutic target to alleviate cardiac hypertrophy.

Accumulated oxidative stress is intimately associated with cellular hypertrophy in the myocardium [3,4,5,6,35]. In line with that, the present results show that GW501516-activated PPARδ significantly inhibits ROS accumulation induced by Ang II in H9c2 cardiomyocytes. Although NADPH oxidase has been implicated as a major source of ROS generation in cardiovascular cells [3,35], we did not directly assess the effects of GW501516-activated PPARδ on NADPH oxidase in the present study. However, we can speculate from the present findings that GW501516-activated PPARδ inhibits ROS production derived from NADPH oxidase by upregulating catalase expression because the inhibitor 3-AT counteracted the suppressive effect of GW501516 on ROS production triggered by Ang II in H9c2 myocytes. Catalase is also known to involved in the suppression of oxidative stress-induced cardiac hypertrophy [27,36]. In fact, the activation of catalase by apelin can prevent oxidative stress-linked cardiac hypertrophy [27]. Furthermore, forkhead transcription factor Foxo3a-mediated transcriptional regulation of catalase inhibits cardiac hypertrophy by modulating ROS generation induced by insulin [37]. These findings therefore suggest that GW501516-activated PPARδ suppresses oxidative stress induced by Ang II through a mechanism involving catalase expression.

PPARδ-mediated induction of the catalase gene in cardiomyocytes is a key event in the suppression of cellular hypertrophy induced by angiotensin II. Catalase, highly conserved among species, is an important antioxidant enzyme that decomposes hydrogen peroxide into water and molecular oxygen [38]. Previous reports indicate that transcriptional regulation of catalase may be complex and involve oxidative stress-related pathways [37,38,39,40,41,42,43,44]. Indeed, the involvement of various transcription factors in this process, such as nuclear factor Y (NF-Y), specificity protein1 (Sp1), fork head box protein O (FoxO3a), CCAAT-enhancer-binding protein β (C/EBP-β), and PPARs, has been demonstrated in multiple cells and tissues [37,39,40,41,42,43,44]. Among these factors, PPARγ, a member of the PPAR family, was originally shown to promote the expression of catalase via a peroxisome proliferator response element (PPRE) in humans and mice [42,43]. A change in catalase expression was also reported in skin receiving a topical application of Wy14643, a specific activator of PPARα, another member of the PPAR family [44]. However, the complete transcriptional regulation of catalase by PPAR member PPARδ has not been elucidated. Accordingly, the present findings clearly show for the first time that PPARδ promotes the expression of catalase in H9c2 cardiomyocytes. 

## 5. Conclusions

The present study show that GW501516-activated PPARδ upregulates the expression of catalase in H9c2 cardiomyocytes, thereby relieving Ang II-induced hypertrophy of myocardial cells by reducing ROS accumulation. The effect of PPARδ ligands on catalase expression may mediate many of the functions of PPARδ, implicating it as a key target for therapeutic intervention in ROS-related cardiovascular disorders such as hypertrophy.

## Data Availability

Data is contained within the article or Supplementary Material.

## Supplementary Materials

The following are available online at https://www.mdpi.com/article/10.3390/antiox10081223/s1. Table S1. Sequence information for primers and siRNA. Figure S1: Effects of GW501516 on the viability of H9c2 cardiomyocytes. Figure S2: Effects of shRNA targeting PPARδ or scrambled control sequences in H9c2 cardiomyocytes. Figure S3: Effects of Ang II on the expression of catalase in H9c2 cardiomyocytes. Figure S4: Effects of siRNA targeting catalase or scrambled control sequences in H9c2 cardiomyocytes.
