# Supplementary material for: Catalase Mediates the Inhibitory Actions of PPARδ against Angiotensin II-Triggered Hypertrophy in H9c2 Cardiomyocytes
Source: Antioxidants (Basel). 2021 Jul 29;10(8):1223. doi: 10.3390/antiox10081223 (PMC8388952; doi:10.3390/antiox10081223)
Supplement: Supplementary file 1 [file antioxidants-10-01223-s001.zip › antioxidants-1296101-supplementary.pdf]

Table S1. Sequence information for primers and siRNA

SiRNA	
scrambled nonspecific sequences	5’-UGGCUAUGGCUCACACACC(dTdT)-3’
rat catalase siRNA	5’-GGUGUGUGAGCCAUAGCCA(dTdT)-3’
Primers for pGL3-catalase luciferase reporter plasmid	
catalase promoter region	Forward:5’-TTGGTACCAGCTCATGCTATGGCCATCA-3’<br>Reverse: 5’-AATCTGTGTCAGCCATAGCGTGCG-3’
Primers for real-time PCR	
Catalase (NM_012520.2)	Forward: 5’-CAAGATGTGGTTTTCACCGACGAGA-3’<br>Reverse: 5’- ATCGTGGGTGACCTCAAAGTATCC-3’
atrial natriuretic peptide (ANP, NM_012612.2)	Forward: 5’- ATCTGATGGATTTCAAGAACC-3’<br>Reverse: 5’-CTCTGAGACGGGTTGACTTC-3’
B-type natriuretic peptide (BNP, NM_031545.1)	Forward: 5’-ACAATCCACGATGCAGAAGCT-3’<br>Reverse: 5’- GGGCCTTGGTCC TTTGAGA-3’
glyceraldehyde-3-phosphate dehydrogenase (GAPDH, NM_017008.4)	Forward: 5’-CATGGCCTTCCGTGTTCCTA-3’<br>Reverse: 5’-CCTGCTTCACCACCT TCTTGAT-3’

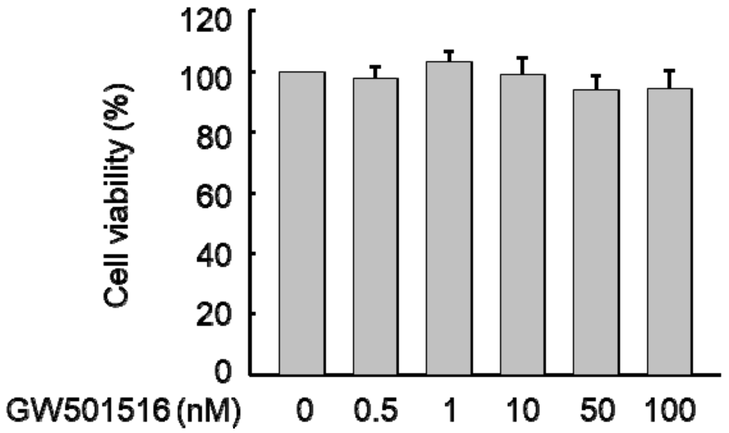

Fig. S1. Effects of GW501516 on the viability of H9c2 cardiomyocytes. Cells were treated for 24 h with various concentrations (0, 0.5, 01, 10, 50, and 100 nM) of GW501516. Cell viability was analyzed by MTT assay. Results are expressed as means ± standard error (SE) (n = 4). Cell viability was not affected at the concentrations of GW501516 used in this study.

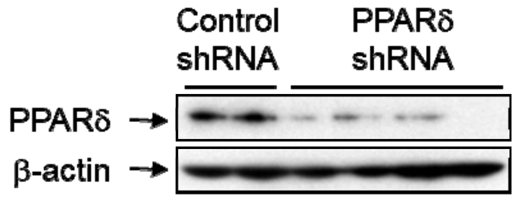

Fig. S2. Effects of shRNA targeting PPARδ or scrambled control sequences in H9c2 cardiomyocytes. Cells were transduced with plasmids harboring the indicated shRNA and selected under 2 μg/ml hygromycin. The efficiency of knockdown was verified by western blotting.

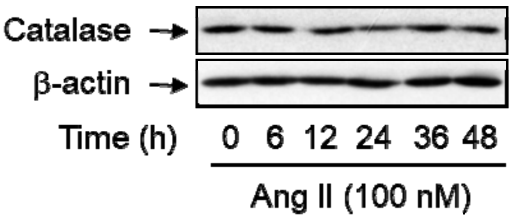

Fig. S3. Effects of Ang II on the expression of catalase in H9c2 cardiomyocytes. Cells were exposed to 100 nM Ang II for the indicated times. The expression of catalase was analyzed by western blotting.

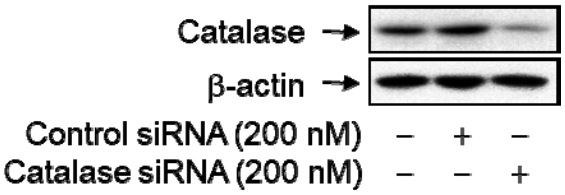

Fig. S4. Effects of siRNA targeting catalase or scrambled control sequences in H9c2 cardiomyocytes. Cells were transfected with the indicated siRNA for 48 h, and the efficiency of siRNA was verified by western blotting.
